# Combined effects of cumulative triglyceride-glucose and blood pressure on stroke in middle-aged and older Chinese adults: a longitudinal analysis

**DOI:** 10.7189/jogh.16.04113

**Published:** 2026-05-22

**Authors:** Xinyue Wang, Wen Zheng, Xiangqian Jin, Hongzhi Liu, Jiheng Hao, Liyong Zhang, Yuanyuan Gu, Haifeng Hou, Wei Wang

**Affiliations:** 1 School of Public Health, Shandong First Medical University & Shandong Academy of Medical Sciences, Jinan, China; 2 Department of Sanatorium, Shandong Provincial Taishan Hospital, Taian, China; 3 Department of Neurosurgery, Liaocheng People’s Hospital, Liaocheng, China; 4 Department of pharmacy, The Affiliated Taian City Central Hospital of Qingdao University, Taian, China; 5 Nutrition and Health Innovation Research Institute & School of Medical and Health Sciences, Edith Cowan University, Joondalup, Western Australia, Australia; 6 Institute of Glycomics, Shantou University Medical College, Shantou, China

## Abstract

**Background:**

The triglyceride-glucose (TyG) index has increasingly been recognised an indicator for stroke risk. We aimed to explore the relationship between TyG and blood pressure (BP) and the combined effects of cumulative TyG (CumTyG) and cumulative BP (CumBP) on stroke risk in a cohort of Chinese middle-aged and older adults.

**Methods:**

We enrolled 5138 participants from the 2011–2018 waves of the China Health and Retirement Longitudinal Study. Cumulative TyG and BP exposures were recorded between 2011 and 2015, and stroke incidence was then followed up until 2018. We conducted a cross-lagged panel analysis to address the temporal and directional relationships between TyG and BP, used logistic regression analysis to determine the effects of CumTyG and CumBP on stroke onset, and employed three machine learning methods to identify the key risk factors and develop a predictive model for stroke.

**Results:**

High TyG levels in 2011 predicted higher systolic BP (SBP) in 2015 (*β1* = 0.026; 95% confidence interval (CI) = 0.001–0.051), and high systolic BP (SBP) in 2011 predicted higher TyG in 2015 (*β2* = 0.028; 95% CI = 0.003–0.053). We observed analogous results in the cross-lag between TyG and diastolic BP (DBP). Compared with individuals in lower CumTyG (<8.62) and cumulative SBP (CumSBP<140 mm Hg), those with higher CumTyG and CumSBP had an increased stroke risk (adjusted odds ratio (aOR) = 1.709; 95% CI = 1.212–2.410). Logistic regression analysis showed similar effects of CumTyG and cumulative DBP (CumDBP) on stroke onset. Seven refined consensus factors were selected to develop the predictive model, by which the area under the receiver operating characteristic curve for prediction of stroke was 0.681 (95% CI = 0.647–0.715) in training and 0.631 (95% CI = 0.573–0.689) in validation sets.

**Conclusions:**

Our findings are indicative of a bidirectional relationship between TyG and BP. Sustained exposure to high levels of TyG index and BP may increase stroke risk.

Stroke has become the second leading cause of mortality and the third cause of disability globally [1,2]. leading to approximately 6.55 million (11.6%) deaths in 2019 [3]. In China, stroke has emerged as the foremost cause of death and disability, with approximately 4.09 million new cases diagnosed in 2021 [3–5].

Hypertension is the major risk factor and a key prognostic indicator of stroke [6–8]. In this context, insulin resistance (IR), typically defined as a reduction in sensitivity and/or responsiveness to the metabolic effects of insulin, impedes glucose utilisation and is closely related to cardiovascular disorders and strokes risk [9–11]. These two distinct pathological conditions – IR and hypertension – have a common molecular basis. Insulin exerts significant vascular actions, which involve stimulating endothelial cells to generate nitric oxide by activating the phosphatidylinositol 3-kinase pathway, resulting in vasodilation [12]. In the state of IR, the mitogen-activated protein kinase pathway is up-regulated, while the activation of the phosphatidylinositol 3-kinase pathway is disturbed, thereby promoting vasoconstriction and hypertension [13]. Furthermore, in hypertensive patients, up-regulation of mineralocorticoid receptor-mediated signal transduction, along with stimulation of the renin-angiotensin-aldosterone system, induce increased production of reactive oxygen species and oxidative stress, which further aggravates IR [14].

The triglyceride-glucose (TyG) index, a composite parameter of fasting blood glucose (FBG) and triglyceride (TG), has emerged as a convincing surrogate indicator of IR [15,16]. Abnormal TyG levels reflect dysregulation in both glucose and lipid metabolism; therefore, focusing on TyG, rather than glucose alone, allows us to conduct a more holistic assessment of dysmetabolism [17]. More importantly, persistently high TyG levels with inadequate metabolic control have been found to predict an increased risk of stroke [18,19]. Yet while this evidence points to the existence of relationships between TyG and blood pressure (BP) and stroke, few studies have explored the role of sustained higher TyG and BP in the development of stroke. This study aimed to elucidate the temporal and directional relationships between TyG and BP, and assess the combined effects of sustained TyG and BP on stroke within a cohort of Chinese middle-aged and elderly adults. In addition, we developed a predictive model for stroke using three machine learning algorithms to investigate the integrative predictive value of these and traditional risk factors.

## METHODS

### Study participants

We retrieved data from four waves (2011, 2013, 2015, and 2018) of China Health and Retirement Longitudinal Study (CHARLS), which is a national prospective cohort study carried out among adults aged 45 years and above [20]. The CHARLS used complex stratified sampling to recruit participants from both rural and urban areas across China, covering 150 counties or districts in 28 provinces [21]. The first wave of CHARLS was launched in 2011. The participants were subsequently followed up in 2013, 2015, and 2018.

Participants were excluded if they had incomplete data in systolic BP (SBP), diastolic BP (DBP), TG or FBG in 2011 or 2015; were younger than 45 years; lacked baseline data; or had a history of stroke in or before 2015. We conducted the cumulative exposure analysis on participants with complete data at both 2011 and 2015 follow-up, ensuring that the indices reflect within-person changes over time.

### Measurement of exposures and outcomes

In the CHARLS, trained interviewers recorded the following sociodemographic and health-related information using a standardised questionnaire: age, sex, educational level (*i.e.* primary school and below, secondary school, and college education and above), marital status, smoking status (never smoking, current smoker and former smoker), alcohol drinking (never drinking, current drinker and former drinker), chronic diseases history (hypertension, diabetes, dyslipidaemia and heart disease), and medications. Trained health examiners also measured BP, height, and body weight.

Investigators from the Chinese Centre for Disease Control and Prevention collected venous blood samples, with FBG, TG, total cholesterol (TC), high-density lipoprotein cholesterol (HDL), low-density lipoprotein cholesterol (LDL), and glycosylated haemoglobin A1c (HbA1c) being examined. We then calculated the TyG index by the formula: ln (TG (mg/dl) × FBG (mg/dl)/2) [22] and computed the time-weighted cumulative TyG index (CumTyG) using the formula: ((TyG_2011_+TyG_2015_)/2 × (visit 2011–2015))/(visit 2011–2015). Similarly, we computed the time-weighted cumulative SBP (CumSBP) and cumulative DBP (CumDBP) using the same method [23].

Body mass index (BMI) was calculated as weight (in kilograms) divided by height (in meters) squared. Hypertension was defined as a BP ≥140/90 mm Hg [24,25], a self-reported history of hypertension, or any use of antihypertensive treatments. Dyslipidemia was defined as meeting any one of the following criteria: having any abnormal blood lipids (TG ≥150 mg/dL, TC ≥240 mg/dL, HDL <40 mg/dL, or LDL ≥160 mg/dL), a self-reported history of dyslipidaemia, or taking lipid-lowering therapy [26]. Diabetes was defined as a FBG ≥7.0 mmol/L, a HbA1c ≥6.5% [27], a self-reported history of diabetes, or the use of antidiabetic treatment.

As the primary outcome of this study, we considered the onset of stroke recorded by the CHARLS team during follow-up, taking into account the current use of anti-stroke medication or a self-reported history of stroke.

### Statistical analysis

We assessed the normality of the distribution for continuous variables using the Shapiro–Wilk test and, due to their non-normal distribution, presented them as medians and interquartile ranges and compared them using the Kruskal–Wallis test. We presented categorical variables as frequencies and percentages, and compared them between groups using the chi-squared test.

We employed a standard cross-lagged panel model that encompassed both autoregressive and cross-lagged paths to investigate the bidirectional relationship between TyG and BP between 2011 and 2015. This analysis assessed the mutual influence between the two variables over time after adjustment for the determinants at baseline, and then quantified the strength of the temporal association [28,29]. Here, we standardised TyG and BP values to a mean of 0 and a standard deviation of 1 to facilitate the comparison of cross-lagged path coefficients across different measurement scales.

We then used logistic regression to evaluate the association between TyG and BP and stroke, calculating odds ratios (ORs) and 95% confidence intervals (CIs) as outputs. To quantify the multiplicative interactions, we additionally included a product term of CumBP (including CumSBP and CumDBP) and CumTyG. *P*-values for interaction were evaluated using likelihood ratio tests and interaction terms. To evaluate the combined effects of CumTyG and CumBP on stroke risk, we allocated participants into four groups based on the clinical threshold for hypertension (≥ 140/90 mmHg *vs*. <140/90 mmHg) and the median CumTyG value (≥ median *vs*. < median). We then used logistic regression to evaluate the association between these four-group classifications and stroke risk We also performed *post-hoc* subgroup analyses, where we stratified participants by sex, age (<60 *vs*. ≥60 years), BMI (<24 *vs*. ≥24 kg/m^2^), and the presence or absence of hypertension, diabetes, dyslipidaemia, and heart disease. We used false discovery rate (FDR) correction to correct for multiple comparisons.

Lastly, we utilised three machine learning algorithms – the elastic net (ENET) model, the Boruta algorithm, and eXtreme Gradient Boosting (XGBoost), to screen significant indicators: baseline data (*i.e.* age, sex, BMI, education level, smoking and alcohol drinking status, history of hypertension, diabetes, heart disease and dyslipidaemia, antihypertensive treatments, antidiabetic treatments, and lipid-lowering treatments) as well as two four-category variables based on the clinical threshold for hypertension (≥140/90 mmHg *vs*. <140/90 mmHg) and the median CumTyG value (≥ median *vs*. < median). We intersected select features from each machine learning algorithm using a Venn diagram to identify a refined set of consensus signatures. Subsequently, we developed a predictive model for stroke using the consensus factors. The predictive value of the model was evaluated using receiver operating characteristic analysis, with calculation of the area under the curve (AUC) and 95% CI. The Hosmer-Lemeshow test was employed to evaluate model calibration.

We performed all statistical analyses with *R*, version 4.3.1 (R Foundation for Statistical Computing, Vienna, Austria), SPSS, version 27.0 (IBM Corporation, Chicago, Illinois, USA) and Mplus, version 8.6 (Muthén & Muthén, Los Angeles, California, USA). We report our study aligns with the GRABDROP guidelines (Table S1 in the **Online Supplementary Document**).

## RESULTS

**Figure 1:**
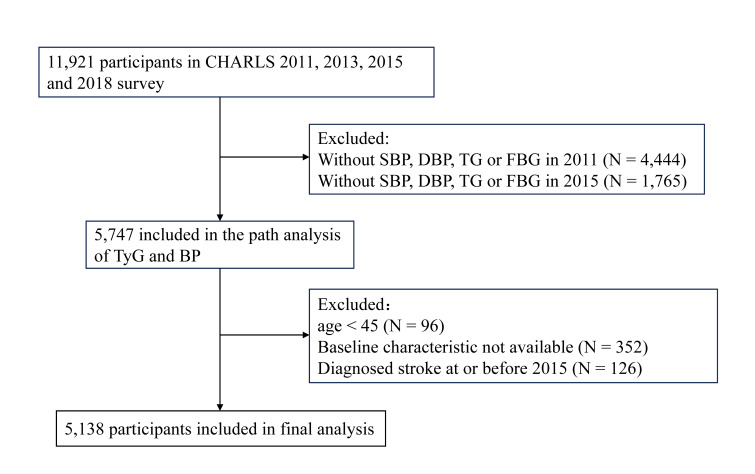
Flowchart of participants. BP – blood pressure, CHARLS – China Health and Retirement Longitudinal Study, DBP – diastolic blood pressure, FBG – fasting blood glucose, SBP – systolic blood pressure, TG – triglyceride, TyG – triglyceride-glucose index.

We initially enrolled 11 921 participants from the four waves of CHARLS, among whom 6783 were excluded due to having incomplete systolic BP, diastolic BP (DBP), TG or FBG in 2011 (n = 4444) and 2015 (n = 1765); being younger than 45 years (n = 96); 3); lacking baseline data (n = 352); having a history of stroke in or before 2015 (n = 126). Finally, 5138 stroke-free respondents were retained for analysis (**Figure 1**). Finally, we retained a sample of 5138 participants (2287 men and 2851 women) with a median age of 58.00 years (Table S2 in the **Online Supplementary Document**).

### Correlation between TyG and BP

After adjusting for age, sex, education level, smoking status, alcohol drinking status, BMI, TC, antihypertensive treatments, antidiabetic treatments, and lipid-lowering treatments, we observed a significant positive correlation between TyG and SBP from 2011 to 2015 (correlation coefficient (*r*) = 0.061~0.063, *P* < 0.001) and between TyG and DBP from 2011 to 2015 (*r* = 0.057~0.068, *P* < 0.001) (Tables S3 and S4 in the **Online Supplementary Document**).

### Cross-lagged path analysis of the relationships of TyG with SBP and DBP

**Figure 2:**
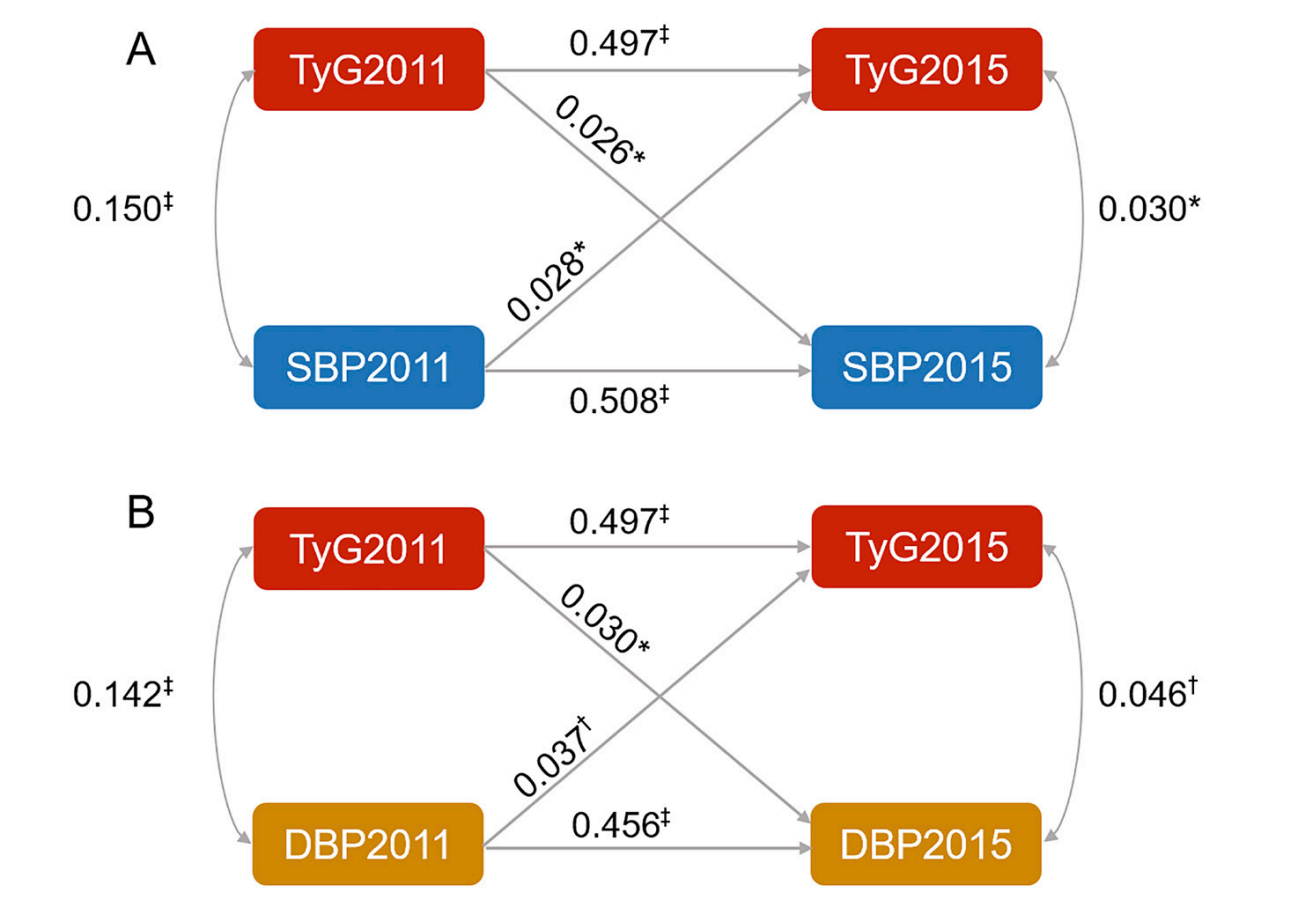
Cross-lagged standard regression coefficients of TyG and BP. **Panel A.** Cross-lagged standard regression coefficients of TyG and SBP. **Panel B.** Cross-lagged standard regression coefficients of TyG and DBP. The cross-lagged model was developed adjusted for age, sex, education level, smoking status, alcohol drinking status, BMI, TC, antihypertensive treatments, antidiabetic treatments, and lipid-lowering treatments. *P < 0.05. †*P* < 0.01, ‡*P* < 0.001. BMI – body mass index, BP – blood pressure, DBP – diastolic blood pressure, SBP – systolic blood pressure, TC – total cholesterol, TyG – triglyceride-glucose index.

After adjusting for potential confounding factors, TyG in 2011 was positively associated with SBP in 2015 (*β1* = 0.026; 95% CI = 0.001–0.051, *P* = 0.044). Meanwhile, SBP in 2011 was correlated with TyG in 2015 (*β2* = 0.028; 95% CI = 0.003–0.053, *P* = 0.027) (**Figure 2**, Panel A; Table S4 in the **Online Supplementary Document**). We also found that TyG in 2011 was positively associated with DBP in 2015 (*β*1 = 0.030; 95% CI = 0.004–0.056, *P* = 0.023), and DBP in 2011 was positively correlated with TyG in 2015 (*β*2 = 0.037; 95% CI = 0.013–0.061, *P* = 0.002) (**Figure 2**, Panel B; Table S6 in the **Online Supplementary Document**).

### Association between cumulative TyG and BP and stroke

CumTyG, CumSBP and CumDBP were independent risk factor for stroke, with adjusted ORs (aORs) of 1.275 (95% CI = 1.039–1.565), 1.014 (95% CI = 1.008–1.021), and 1.021 (95% CI = 1.010–1.033), respectively (**Table 1**). Notably, our logistic regression analysis showed a significant interaction between CumTyG and CumSBP (OR = 1.002; 95% CI = 1.001–1.002, *P* < 0.001) and between CumTyG and CumDBP (OR = 1.002; 95% CI = 1.001–1.004, *P* < 0.001).

**Table 1:** Logistic regression analysis on association between CumTyG and CumBP and stroke incidence*

	Unadjusted model		Model 1		Model 2		Model 3	
**Variables**	**OR (95% CI)**	***P*-value**	**OR (95% CI)**	***P*-value**	**OR (95% CI)**	***P*-value**	**OR (95% CI)**	***P*-value**
TyG in 2011	1.470 (1.257–1.719)	<0.001	1.494 (1.275–1.751)	<0.001	1.387 (1.175–1.637)	<0.001	1.257 (1.058–1.495)	0.009
SBP in 2011	1.016 (1.011–1.020)	<0.001	1.014 (1.009–1.019)	<0.001	1.012 (1.007–1.017)	<0.001	1.009 (1.003–1.014)	0.002
DBP in 2011	1.019 (1.011–1.028)	<0.001	1.021 (1.013–1.030)	<0.001	1.018 (1.009–1.079)	<0.001	1.013 (1.004–1.022)	0.006
TyG in 2015	1.378 (1.168–1.626)	<0.001	1.424 (1.203–1.685)	<0.001	1.285 (1.076–1.533)	0.005	1.145 (0.951–1.377)	0.152
SBP in 2015	1.020 (1.015–1.025)	<0.001	1.018 (1.012–1.023)	<0.001	1.016 (1.011 1.022)	<0.001	1.013 (1.007–1.018)	<0.001
DBP in 2015	1.020 (1.012–1.028)	<0.001	1.022 (1.014–1.031)	<0.001	1.020 (1.012–1.029)	<0.001	1.017 (1.008,1.026)	<0.001
CumTyG	1.568 (1.309–1.879)	<0.001	1.618 (1.347–1.945)	<0.001	1.458 (1.200–1.770)	<0.001	1.275 (1.039–1.565)	0.020
CumSBP	1.023 (1.017–1.028)	<0.001	1.020 (1.015–1.026)	<0.001	1.019 (1.013–1.025)	<0.001	1.014 (1.008–1.021)	<0.001
CumDBP	1.027 (1.017–1.037)	<0.001	1.031 (1.020–1.041)	<0.001	1.027 (1.017,1.038)	<0.001	1.021 (1.010–1.033)	<0.001

BMI – body mass index, BP – blood pressure, CI – confidence interval, CumDBP – cumulative diastolic blood pressure, CumSBP – cumulative systolic blood pressure, CumTyG – cumulative triglyceride-glucose index, OR – odds ratio

*Model 1: adjusted for age and sex. Model 2: adjusted for age, sex, education level, smoking status, alcohol drinking, and BMI. Model 3: adjusted for factors in model 2 and history of hypertension, dyslipidaemia, diabetes, heart disease, antihypertensive treatments, antidiabetic treatments, and lipid-lowering treatments.

### Combined effects of cumulative TyG and BP on stroke risk

Participants were allocated into four groups based on the clinical threshold for hypertension (SBP ≥140 mmHg) and the median CumTyG value (8.62): CumTyG <8.62, CumSBP <140 mmHg; CumTyG ≥8.62, CumSBP <140 mmHg; CumTyG <8.62, CumSBP ≥140 mmHg; CumTyG ≥8.62, CumSBP ≥140 mmHg. There were 330 (6.4%) participants with an onset of stroke during our follow-up between 2011 and 2018. Compared to the individuals in group 1, those in groups 2, 3 and 4 had increased risks for stroke, with the crude ORs of 1.377 (95% CI = 1.032–1.837), 1.784 (95% CI = 1.226–2.594), and 2.750 (95% CI = 2.023–3.739), respectively. After adjusting for covariates, the aORs were 1.145 (95% CI = 0.845–1.550), 1.275 (95% CI = 0.861–1.888), and 1.709 (95% CI = 1.212–2.410), respectively (**Table 2**; Figure S1, Panel A in the **Online Supplementary Document**).

**Table 2:** Logistic regression analysis for the association between co-exposure to CumTyG and CumSBP on stroke incidence*

		Unadjusted model		Model 1		Model 2		Model 3	
**Variables**	**Event/total**	**OR (95% CI)**	***P*-value**	**OR (95% CI)**	***P*-value**	**OR (95% CI)**	***P*-value**	**OR (95% CI)**	***P*-value**
CumTyG<8.62, CumSBP<140 mm Hg	88/1975	ref		ref		ref		ref	
CumTyG≥8.62, CumSBP<140 mm Hg	109/1807	1.377 (1.032–1.837)	0.030	1.411 (1.055–1.887)	0.020	1.263 (0.938–1.701)	0.124	1.145 (0.845–1.550)	0.382
CumTyG<8.62, CumSBP≥140 mm Hg	44/573	1.784 (1.226–2.594)	0.002	1.585 (1.084–2.316)	0.017	1.497 (1.022–2.193)	0.038	1.275 (0.861–1.888)	0.226
CumTyG≥8.62, CumSBP≥140 mm Hg	89/783	2.750 (2.023–3.739)	<0.001	2.546 (1.864–3.477)	<0.001	2.174 (1.574–3.005)	<0.001	1.709 (1.212–2.410)	0.002

BMI – body mass index, CI – confidence interval, CumSBP – cumulative systolic blood pressure, CumTyG – cumulative triglyceride-glucose index, OR – odds ratio, ref – reference

*Model 1: adjusted for age and sex. Model 2: adjusted for age, sex, education level, smoking status, alcohol drinking, and BMI. Model 3: adjusted for factors in model 2 and history of hypertension, dyslipidaemia, diabetes, heart disease, antihypertensive treatments, antidiabetic treatments, and lipid-lowering treatments.

To specifically evaluate the role of DBP, participants were also divided into four groups based on the clinical thresholds for hypertension (DBP ≥90 mmHg) and the median CumTyG value (8.62): CumTyG <8.62, CumDBP <90 mmHg; CumTyG ≥8.62, CumDBP <90 mmHg; CumTyG <8.62, CumDBP ≥90 mmHg; CumTyG ≥8.62, CumDBP ≥90 mmHg. We obtained similar results regarding the association between CumTyG and CumDBP and stroke. Compared to the participants in group 1 (CumTyG <8.62 and CumDBP<90 mmHg), the aORs for developing stroke in the groups 2, 3, and 4 were 1.165 (95% CI = 0.897–1.512), 1.080 (95% CI = 0.576–2.026), and 1.785 (95% CI = 1.169–2.725), respectively (**Table 3**; Figure S1, Panel B in the **Online Supplementary Document**).

**Table 3:** Logistic regression analysis for the association between co-exposure to CumTyG and CumDBP on stroke incidence*

		Unadjusted model		Model 1		Model 2		Model 3	
**Variables**	**Event/total**	**OR (95% CI)**	***P*-value**	**OR (95% CI)**	***P*-value**	**OR (95% CI)**	***P*-value**	**OR (95% CI)**	***P*-value**
CumTyG<8.62, CumDBP<90 mm Hg	120/2364	ref		ref		ref		ref	
CumTyG≥8.62, CumDBP<90 mm Hg	162/2277	1.432 (1.123–1.827)	0.004	1.463 (1.143–1.872)	0.003	1.305 (1.012–1.682)	0.040	1.165 (0.897–1.512)	0.251
CumTyG<8.62, CumDBP≥90 mm Hg	12/184	1.305 (0.707–2.409)	0.395	1.420 (0.767–2.629)	0.264	1.338 (0.721–2.484)	0.356	1.080 (0.576–2.026)	0.810
CumTyG≥8.62, CumDBP≥90 mm Hg	36/313	2.430 (1.641–3.600)	<0.001	2.681 (1.803–3.985)	<0.001	2.248 (1.496–3.379)	<0.001	1.785 (1.169–2.725)	0.007

BMI – body mass index, CI – confidence interval, CumDBP – cumulative diastolic blood pressure, CumTyG – cumulative triglyceride-glucose index, OR – odds ratio, ref – reference

*Model 1: adjusted for age and sex. Model 2: adjusted for age, sex, education level, smoking status, alcohol drinking, and BMI. Model 3: adjusted for factors in model 2 and history of hypertension, dyslipidaemia, diabetes, heart disease, antihypertensive treatments, antidiabetic treatments, and lipid-lowering treatments.

### Stratified analysis of stroke risk based on CumTyG and CumBP

**Figure 3:**
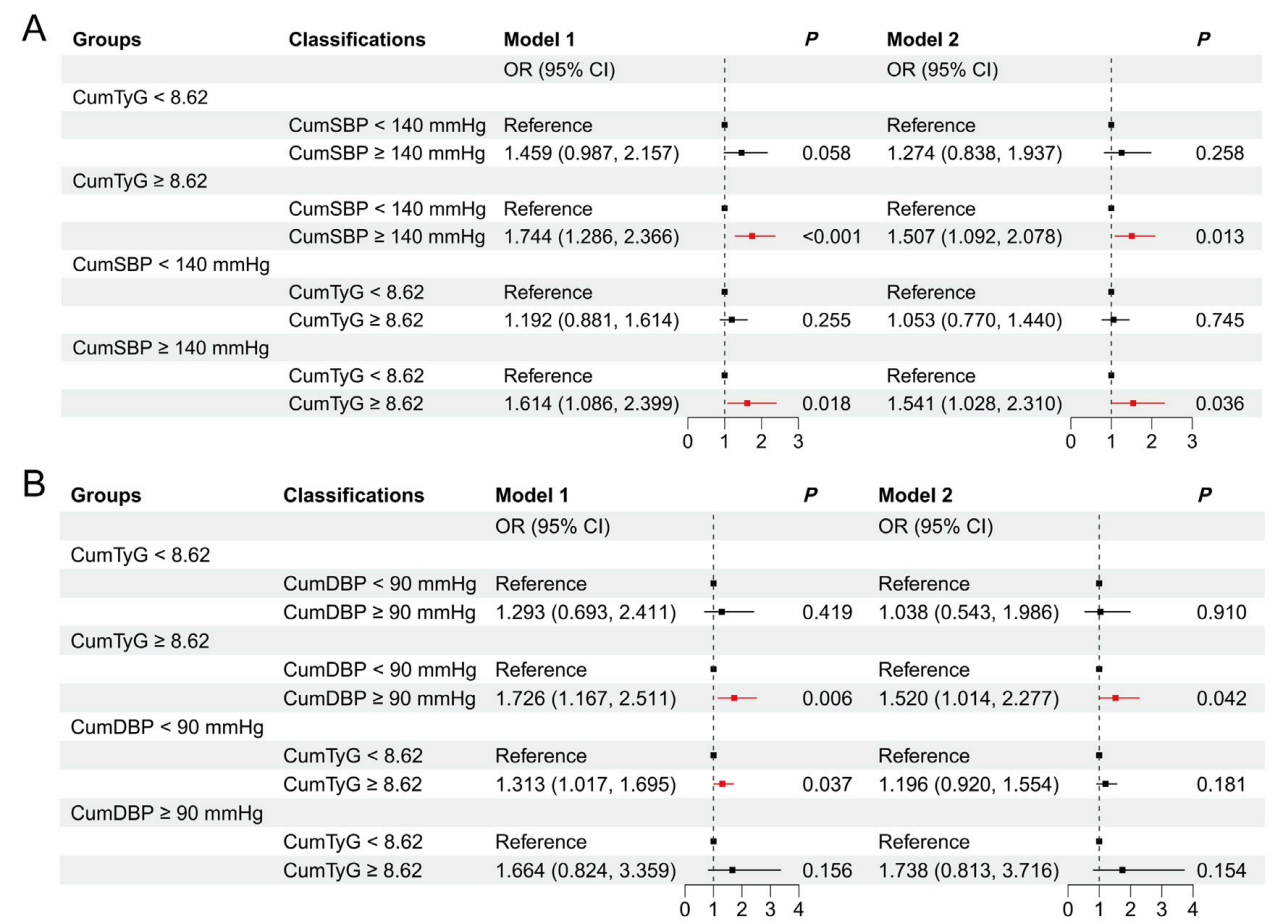
Stratified analysis of stroke risk based on CumTyG and CumBP. **Panel A.** Stratified analysis of stroke risk based on CumTyG and CumSBP. **Panel B.** Stratified analysis of stroke risk based on CumTyG and CumDBP. Model 1: adjusted for age, sex, education level, smoking status, alcohol drinking status, and BMI. Model 2: adjusted for covariates in model 1 and history of hypertension, dyslipidaemia, diabetes, heart disease, antihypertensive treatments, antidiabetic treatments, and lipid-lowering treatments. BMI – body mass index, CI – confidence interval, CumDBP – cumulative diastolic blood pressure, CumSBP – cumulative systolic blood pressure, CumTyG – cumulative triglyceride-glucose index, OR – odds ratio.

For participants with CumSBP <140 mmHg, higher CumTyG was not significantly associated with stroke risk (aOR = 1.053; 95% CI = 0.770–1.440). However, for those with CumSBP ≥140 mmHg, a higher CumTyG level was significantly linked to higher risk for stroke (aOR = 1.541; 95% CI = 1.028–2.310). Similarly, for participants with CumTyG <8.62, a higher CumSBP level was not significantly associated with stroke risk (aOR = 1.274; 95% CI = 0.838–1.937). In contrast, for those with CumTyG ≥8.62, a higher CumSBP level was significantly associated with an increased stroke risk (aOR = 1.507; 95% CI = 1.092–2.078) (**Figure 3**, Panel A; Table S7 in the **Online Supplementary Document**). We observed similar associations in our analyses involving CumTyG and CumDBP (<90 mmHg and ≥90 mmHg) (**Figure 3**, Panel B; Table S8 in the **Online Supplementary Document**).

### Subgroup analysis

Among participants who were males, aged ≥60 years, had a BMI<24, and had no history of hypertension, diabetes, dyslipidaemia, or heart disease, individuals with both higher CumTyG and CumSBP had an increased risk of stroke compared to those with lower levels of both risk factors (Table S9 in the **Online Supplementary Document**). Among participants who were males, aged <60 years, and had no comorbid dyslipidaemia or heart disease, individuals with both higher CumTyG and CumDBP had an increased stroke risk compared to those with lower levels of both risk factors (Table S10 in the **Online Supplementary Document**).

### Development of predictive model for stroke

We divided all participants into a training set (n = 3596) and a validation set (n = 1542) using a 7:3 ratio. Nine key factors (CumTyG-CumSBP groups, CumTyG-CumDBP groups, age, sex, BMI, smoking status, alcohol drinking status, history of hypertension, and antihypertensive treatments) were selected by the Boruta algorithm, 10 (CumTyG-CumSBP groups, CumTyG-CumDBP groups, age, sex, BMI, education level, smoking status, alcohol drinking status, history of hypertension, and antihypertensive treatments) by the XGBoost algorithm, and (CumTyG-CumSBP groups, CumTyG-CumDBP groups, age, BMI, smoking status, history of hypertension, diabetes, dyslipidaemia and heart disease, antihypertensive treatments and lipid-lowering treatments) by the ENET algorithm (Figure S2 in the **Online Supplementary Document**). We derived seven refined consensus factors (CumTyG-CumSBP groups, CumTyG-CumDBP groups, age, BMI, smoking status, history of hypertension and antihypertensive treatments) following the intersections depicted in the Venn diagram (Figure S3 in the **Online Supplementary Document**). Using these seven consensus factors, we constructed a predictive model for stroke. We used receiver operating characteristic curves to assess the performance of the predictive model, where the AUC was 0.681 (95% CI = 0.647–0.715) in the training and 0.631 (95% CI = 0.573–0.689) in validation sets (Figure S4 in the **Online Supplementary Document**). The Hosmer-Lemeshow test also demonstrated appropriate model calibration, with *P*-values of 0.228 in the training sets and 0.896 in the validation sets.

## DISCUSSION

Our findings indicate that higher TyG and BP levels are associated with increased risk for stroke. Specificaly, individuals with a high level of TyG and BP, as expressed by CumTyG, CumSBP, and CumDBP, may be exposed to a higher risk for stroke. We also observed a significant interaction between CumTyG and CumBP, which linked to stroke risk. In addition, we noted a bidirectional correlation between TyG and BP, indicating that higher TyG was correlated with higher BP and *vice versa*.

TyG, one of metabolic indicators, has been acknowledged as a convenient substitutive index for evaluating IR [30,31]. Previous studies have identified an association between TyG and BP [32], reporting that one unit rise in TyG increased 1.93 mm Hg in SBP and 1.78 mm Hg in DBP [33]. As is known, IR induces over-activation of renin-angiotensin system and then increases vascular resistance, resulting in the elevation of BP [14,34–36]. In addition, higher BP has been correlated with inflammatory responses and oxidative stress that interfere with insulin receptor-mediated signalling [37,38]. These components influence the differentiation and metabolism of adipose cells, augmenting the release of free fatty acids, thereby further exacerbating IR [39–41]. However, few studies have explored the bidirectional association between TyG and BP in longitudinal cohorts. Here, we identified the temporal and directional relationships of TyG with both SBP and DBP using a cross-lagged design. After adjusting for covariates, *i.e.* demographics, medical history, and clinical characteristics, the association remained robust. The comparable magnitude of the cross-lagged path coefficients indicates a mutual reinforcement between TyG and BP, rather than a unidirectional causal pathway. For longitudinal exposure assessment, we constructed time-weighted cumulative indices (CumTyG, CumSBP, and CumDBP), allowing us to address a key limitation of using a single baseline measurement, which may fail to capture the sustained exposure relevant to chronic disease risk. By integrating repeated measurements over time, cumulative indices provide a more robust measure of an individual’s long-term exposure burden, reflecting both the intensity and duration of exposure [23,42,43].

A community-based, prospective study has shown that hypertensive patients with a maintained higher level of TyG have a higher risk for stroke than those with a normal TyG level [44]. Although the underlying mechanisms remain to be determined, the relationship between CumTyG and CumBP and the risk of stroke is potentially linked to IR. In detail, IR increases the risks of chronic metabolic diseases, such as diabetes, hypertension, and dyslipidaemia, which are key risk factors for stroke [45]. It also affects platelet activation, adhesion, and aggregation, accelerating arterial stenosis or occlusion and thereby increasing stroke risk [46–48]. In addition, arterial stiffness is a crucial component of the pathogenesis of stroke [49]. Studies have demonstrated that an elevated TyG level shows a significant correlation with arterial stiffness in hypertensive population [50,51]. Therefore, exploring the combined effect of TyG index and BP has significant implications for prophylaxis and management of stroke. Here, we observed that high TyG and BP were not only independently linked to stroke risk, but also worked in an interaction manner. Furthermore, the sustained exposure to higher TyG and BP was associated with a heightened risk of stroke.

Subsequently, we developed a predictive model for stroke by integrating CumTyG-BP and traditional risk factors using machine learning algorithms. The model achieved a modest predictive ability, with an AUC of 0.681 (95% CI = 0.647–0.715) in the training set and 0.631 (95% CI = 0.573–0.689) in the validation set. The performance of our model compared favourably with established single-measurement TyG indices from the same CHARLS cohort, including TyG-BMI (AUC = 0.593; 95% CI = 0.573–0.613), TyG-waist circumference (AUC = 0.608; 95% CI = 0.588–0.628), and TyG-waist-to-height ratio (AUC = 0.603; 95% CI = 0.583–0.623) [52]. The performance was also comparable to a CHARLS-based model predicting social participation in stroke, which incorporated 11 sociodemographic and health factors, and reported AUCs of 0.669 (95% CI = 0.631–0.707) in the training set and 0.635 (95% CI = 0.573–0.698) in the validation set [53]. This level of performance reflected the inherent complexity of stroke pathogenesis, which involved a complex interplay of genetic, environmental, and unmeasured clinical determinants not fully captured in our dataset [54]. Despite its limitations, this model substantiates the conceptual utility of combining metabolic and hemodynamic indices for stroke risk prediction and provides a foundational framework for future model refinement.

Our study includes a large and representative national sample, thereby allowing for broad generalisability to the middle-aged and older Chinese population. Moreover, we longitudinally collected TyG and BP information of the participants before the occurrence of stroke to directly evaluate the sustained impact of combined cumulative exposure to TyG and BP on stroke risk. All these aspects provided evidence for the stability and credibility of our conclusions. As far as we are aware, no prior study has explored the effect of the pathway temporally connecting CumTyG and CumBP on stroke risk.

However, we acknowledge several limitations. First, the stroke event was self-reported by the respondents, posing a possibility of reporting error. Explicit medical records for stroke are absent in the CHARLS dataset. However, we note that the large-scale English Longitudinal Study of Ageing, which has a similar methodology to the CHARLS, has demonstrated an acceptable level of consistency between self-reported stroke events and medical records [55]. Second, we excluded participants who had incomplete information on BP or TyG, which may have lead to information bias. Third, our predictive model has not undergone external validation, which limits the assessment of its generalisability and clinical applicability. Finally, even after adjusting for possible confounding variables, a possibility of residual or unassessed confounding cannot be ruled out.

## CONCLUSIONS

In our sample, we noted a bidirectional relationship between TyG and BP, and found that a sustained exposure to high TyG index and BP may increase stroke risk. These observations indicate the importance of maintenance of appropriate TyG and BP levels for the prevention of stroke.

## Additional material


Online Supplementary Document

## References

[1] Safiri S Nejadghaderi SA Karamzad N Carson-Chahhoud K Bragazzi NL Sullman MJM Global, regional, and national cancer deaths and disability-adjusted life-years (DALYs) attributable to alcohol consumption in 204 countries and territories, 1990-2019. Cancer. 2022;128:1840–52. 10.1002/cncr.34111 35239973

[2] Kuriakose D Xiao Z Pathophysiology and Treatment of Stroke: Present Status and Future Perspectives. Int J Mol Sci. 2020;21:7609. 10.3390/ijms21207609 33076218 PMC7589849

[3] GBD 2019 Stroke Collaborators Global, regional, and national burden of stroke and its risk factors, 1990–2019: a systematic analysis for the Global Burden of Disease Study 2019. Lancet Neurol. 2021;20:795–820. 10.1016/S1474-4422(21)00252-0 34487721 PMC8443449

[4] Liu L Chen W Zhou H Duan W Li S Huo X Chinese Stroke Association guidelines for clinical management of cerebrovascular disorders: executive summary and 2019 update of clinical management of ischaemic cerebrovascular diseases. Stroke Vasc Neurol. 2020;5:159–76. 10.1136/svn-2020-000378 32561535 PMC7337371

[5] Tu WJ Wang LD Yan F Peng B Hua Y Liu M China stroke surveillance report 2021. Mil Med Res. 2023;10:33. 37468952 10.1186/s40779-023-00463-x PMC10355019

[6] Cipolla MJ Liebeskind DS Chan SL The importance of comorbidities in ischemic stroke: Impact of hypertension on the cerebral circulation. J Cereb Blood Flow Metab. 2018;38:2129–49. 10.1177/0271678X18800589 30198826 PMC6282213

[7] Diener HC Hankey GJ Primary and Secondary Prevention of Ischemic Stroke and Cerebral Hemorrhage. J Am Coll Cardiol. 2020;75:1804–18. 10.1016/j.jacc.2019.12.072 32299593

[8] Yu JG Zhou RR Cai GJ From Hypertension to Stroke: Mechanisms and Potential Prevention Strategies. CNS Neurosci Ther. 2011;17:577–84. 10.1111/j.1755-5949.2011.00264.x 21951373 PMC6493871

[9] Petrie JR Guzik TJ Touyz RM Diabetes, Hypertension, and Cardiovascular Disease: Clinical Insights and Vascular Mechanisms. Can J Cardiol. 2018;34:575–84. 10.1016/j.cjca.2017.12.005 29459239 PMC5953551

[10] Rochlani Y Pothineni NV Kovelamudi S Mehta JL Metabolic syndrome: pathophysiology, management, and modulation by natural compounds. Ther Adv Cardiovasc Dis. 2017;11:215–25. 10.1177/1753944717711379 28639538 PMC5933580

[11] Kernan WN Inzucchi SE Viscoli CM Brass LM Bravata DM Shulman GI Impaired insulin sensitivity among nondiabetic patients with a recent TIA or ischemic stroke. Neurology. 2003;60:1447–51. 10.1212/01.WNL.0000063318.66140.A3 12743229

[12] Kim JA Montagnani M Koh KK Quon MJ Reciprocal relationships between insulin resistance and endothelial dysfunction: molecular and pathophysiological mechanisms. Circulation. 2006;113:1888–904. 10.1161/CIRCULATIONAHA.105.563213 16618833

[13] Cao Z Wang J Pang N Lin Y Liu X Associations between novel triglyceride-glucose-related indices and the incidence of hypertension among Chinese middle-aged and elderly adults: a nationwide prospective cohort study. Cardiovasc Diabetol. 2025;11:50. 10.1186/s40842-025-00255-3 41457293 PMC12746625

[14] Artunc F Schleicher E Weigert C Fritsche A Stefan N Häring H-U The impact of insulin resistance on the kidney and vasculature. Nat Rev Nephrol. 2016;12:721–37. 10.1038/nrneph.2016.145 27748389

[15] Guerrero-Romero F Simental-Mendía LE González-Ortiz M Martínez-Abundis E Ramos-Zavala MG Hernández-González SO The product of triglycerides and glucose, a simple measure of insulin sensitivity. Comparison with the euglycemic-hyperinsulinemic clamp. J Clin Endocrinol Metab. 2010;95:3347–51. 10.1210/jc.2010-0288 20484475

[16] Irace C Carallo C Scavelli FB De Franceschi MS Esposito T Tripolino C Markers of insulin resistance and carotid atherosclerosis. A comparison of the homeostasis model assessment and triglyceride glucose index. Int J Clin Pract. 2013;67:665–72. 10.1111/ijcp.12124 23758445

[17] Sun Y Ji H Sun W An X Lian F Triglyceride glucose (TyG) index: A promising biomarker for diagnosis and treatment of different diseases. Eur J Intern Med. 2025;131:3–14. 10.1016/j.ejim.2024.08.026 39510865

[18] Wang A Wang G Liu Q Zuo Y Chen S Tao B Triglyceride-glucose index and the risk of stroke and its subtypes in the general population: an 11-year follow-up. Cardiovasc Diabetol. 2021;20:46. 10.1186/s12933-021-01238-1 33602208 PMC7893902

[19] Wu Y Yang Y Zhang J Liu S Zhuang W The change of triglyceride-glucose index may predict incidence of stroke in the general population over 45 years old. Cardiovasc Diabetol. 2023;22:132. 10.1186/s12933-023-01870-z 37296457 PMC10257314

[20] Zhao Y Hu Y Smith JP Strauss J Yang G Cohort Profile: The China Health and Retirement Longitudinal Study (CHARLS). Int J Epidemiol. 2014;43:61–8. 10.1093/ije/dys203 23243115 PMC3937970

[21] Hu Y Peng W Ren R Wang Y Wang G Sarcopenia and mild cognitive impairment among elderly adults: The first longitudinal evidence from CHARLS. J Cachexia Sarcopenia Muscle. 2022;13:2944–52. 10.1002/jcsm.13081 36058563 PMC9745544

[22] Simental-Mendía LE Rodríguez-Morán M Guerrero-Romero F The Product of Fasting Glucose and Triglycerides As Surrogate for Identifying Insulin Resistance in Apparently Healthy Subjects. Metab Syndr Relat Disord. 2008;6:299–304. 10.1089/met.2008.0034 19067533

[23] Wu K Zheng H Wu W Chen G Cai Z Cai Z Temporal relationship between triglyceride-glucose index and blood pressure and their joint cumulative effect on cardiovascular disease risk: a longitudinal cohort study. Cardiovasc Diabetol. 2023;22:332. 10.1186/s12933-023-02058-1 38017521 PMC10685547

[24] Gao K Cao L-F Ma W-Z Gao Y-J Luo M-S Zhu J Association between sarcopenia and cardiovascular disease among middle-aged and older adults: Findings from the China health and retirement longitudinal study. EClinicalMedicine. 2022;44:101264. 10.1016/j.eclinm.2021.101264 35059617 PMC8760427

[25] Zhang M Shi Y Zhou B Huang Z Zhao Z Li C Prevalence, awareness, treatment, and control of hypertension in China, 2004-18: findings from six rounds of a national survey. BMJ. 2023;380:e071952. 10.1136/bmj-2022-071952 36631148 PMC10498511

[26] Zhang L Li S Liu D Gui J Hu J Wang Q The relationship between C-reactive protein-triglyceride-glucose index and cardiovascular disease: insights from the China health and retirement longitudinal study (CHARLS). Cardiovasc Diabetol. 2025;24:410. 10.1186/s12933-025-02960-w 41152893 PMC12560464

[27] Weng J Ji L Jia W Lu J Zhou Z Zou D Standards of care for type 2 diabetes in China. Diabetes Metab Res Rev. 2016;32:442–58. 10.1002/dmrr.2827 27464265 PMC5108436

[28] Zheng DD Swenor BK Christ SL West SK Lam BL Lee DJ Longitudinal Associations Between Visual Impairment and Cognitive Functioning. JAMA Ophthalmol. 2018;136:989–95. 10.1001/jamaophthalmol.2018.2493 29955805 PMC6142982

[29] Wu M Ya Z Mawditt C Liao J Bidirectional association between clustering of health-related behaviours and depression in mid- and older-aged adults: A longitudinal study in China and Japan. J Affect Disord. 2025;376:294–301. 10.1016/j.jad.2024.12.112 39922290

[30] Sánchez-García A Rodríguez-Gutiérrez R Mancillas-Adame L González-Nava V Díaz González-Colmenero A Solis RC Diagnostic Accuracy of the Triglyceride and Glucose Index for Insulin Resistance: A Systematic Review. Int J Endocrinol. 2020;2020:4678526. 10.1155/2020/4678526 32256572 PMC7085845

[31] Navarro-González D Sánchez-Íñigo L Pastrana-Delgado J Fernández-Montero A Martinez JA Triglyceride–glucose index (TyG index) in comparison with fasting plasma glucose improved diabetes prediction in patients with normal fasting glucose: The Vascular-Metabolic CUN cohort. Prev Med. 2016;86:99–105. 10.1016/j.ypmed.2016.01.022 26854766

[32] Lee DH Park JE Kim SY Jeon HJ Park JH Association between the triglyceride-glucose (TyG) index and increased blood pressure in normotensive subjects: a population-based study. Diabetol Metab Syndr. 2022;14:161. 10.1186/s13098-022-00927-5 36309720 PMC9617408

[33] Wang D Li W Zhou M Ma J Guo Y Yuan J Association of the triglyceride-glucose index variability with blood pressure and hypertension: a cohort study. QJM: monthly journal of the Association of Physicians. 2024:117:277–82. 10.1093/qjmed/hcad252 37950450

[34] Endre T Mattiasson I Berglund G Hulthén UL Insulin and renal sodium retention in hypertension-prone men. Hypertension. 1994;23:313–9. 10.1161/01.HYP.23.3.313 8125556

[35] Kalupahana NS Moustaid-Moussa N The renin-angiotensin system: a link between obesity, inflammation and insulin resistance. Obes Rev. 2012;13:136–49. 10.1111/j.1467-789X.2011.00942.x 22034852

[36] Modan M Halkin H Hyperinsulinemia or increased sympathetic drive as links for obesity and hypertension. Diabetes Care. 1991;14:470–87. 10.2337/diacare.14.6.470 1864220

[37] Karaca Ü Schram MT Houben AJHM Muris DMJ Stehouwer CDA Microvascular dysfunction as a link between obesity, insulin resistance and hypertension. Diabetes Res Clin Pract. 2014;103:382–7. 10.1016/j.diabres.2013.12.012 24438874

[38] Zhang Z Zhao L Zhou X Meng X Zhou X Role of inflammation, immunity, and oxidative stress in hypertension: New insights and potential therapeutic targets. Front Immunol. 2023;13:1098725. 10.3389/fimmu.2022.1098725 36703963 PMC9871625

[39] Chapman MJ Sposito AC Hypertension and dyslipidaemia in obesity and insulin resistance: Pathophysiology, impact on atherosclerotic disease and pharmacotherapy. Pharmacol Ther. 2008;117:354–73. 10.1016/j.pharmthera.2007.10.004 18215759

[40] Issa N Lachance G Bellmann K Laplante M Stadler K Marette A Cytokines promote lipolysis in 3T3-L1 adipocytes through induction of NADPH oxidase 3 expression and superoxide production. J Lipid Res. 2018;59:2321–8. 10.1194/jlr.M086504 30317185 PMC6277153

[41] Karpe F Dickmann JR Frayn KN Fatty acids, obesity, and insulin resistance: time for a reevaluation. Diabetes. 2011;60:2441–9. 10.2337/db11-0425 21948998 PMC3178283

[42] Nuotio J Suvila K Cheng S Langén V Niiranen T Longitudinal blood pressure patterns and cardiovascular disease risk. Ann Med. 2020;52:43–54. 10.1080/07853890.2020.1733648 32077328 PMC7877994

[43] Tian X Chen S Zhang Y Zhang X Xu Q Wang P Time course of the triglyceride glucose index accumulation with the risk of cardiovascular disease and all-cause mortality. Cardiovasc Diabetol. 2022;21:183. 10.1186/s12933-022-01617-2 36100896 PMC9472367

[44] Huang Z Ding X Yue Q Wang X Chen Z Cai Z Triglyceride-glucose index trajectory and stroke incidence in patients with hypertension: a prospective cohort study. Cardiovasc Diabetol. 2022;21:141. 10.1186/s12933-022-01577-7 35897017 PMC9331781

[45] Zhao X An X Yang C Sun W Ji H Lian F The crucial role and mechanism of insulin resistance in metabolic disease. Front Endocrinol (Lausanne). 2023;14:1149239. 10.3389/fendo.2023.1149239 37056675 PMC10086443

[46] Trovati M Anfossi G Influence of insulin and of insulin resistance on platelet and vascular smooth muscle cell function. J Diabetes Complications. 2002;16:35–40. 10.1016/S1056-8727(01)00196-9 11872364

[47] Deng XL Liu Z Wang Cl Li Yf, Cai Zy. Insulin resistance in ischemic stroke. Metab Brain Dis. 2017;32:1323–34. 10.1007/s11011-017-0050-0 28634787

[48] Ferreiro JL Gómez-Hospital JA Angiolillo DJ Platelet abnormalities in diabetes mellitus. Diab Vasc Dis Res. 2010;7:251–9. 10.1177/1479164110383994 20921090

[49] Chen Y Shen F Liu J Yang G-Y Arterial stiffness and stroke: de-stiffening strategy, a therapeutic target for stroke. Stroke Vasc Neurol. 2017;2:65–72. 10.1136/svn-2016-000045 28959494 PMC5600012

[50] Wu Z Zhou D Liu Y Li Z Wang J Han Z Association of TyG index and TG/HDL-C ratio with arterial stiffness progression in a non-normotensive population. Cardiovasc Diabetol. 2021;20:134. 10.1186/s12933-021-01330-6 34229681 PMC8262008

[51] Li M Zhan A Huang X Hu L Zhou W Wang T Positive association between triglyceride glucose index and arterial stiffness in hypertensive patients: the China H-type Hypertension Registry Study. Cardiovasc Diabetol. 2020;19:139. 10.1186/s12933-020-01124-2 32948181 PMC7501677

[52] Sun J Meng X Guo L Nian C Li H Huang W Association between modified triglyceride glucose indices and stroke risk in middle-aged and older Chinese adults: a prospective cohort study. Cardiovasc Diabetol. 2025;24:274. 10.1186/s12933-025-02827-0 40640840 PMC12243310

[53] Liu Y Li T Ding L Cai Z Nie S A predictive model for social participation of middle-aged and older adult stroke survivors: the China Health and Retirement Longitudinal Study. Front Public Health. 2024;11:1271294. 10.3389/fpubh.2023.1271294 38283296 PMC10810982

[54] Boehme AK Esenwa C Elkind MSV Stroke Risk Factors, Genetics, and Prevention. Circ Res. 2017;120:472–95. 10.1161/CIRCRESAHA.116.308398 28154098 PMC5321635

[55] Xie W Zheng F Yan L Zhong B Cognitive Decline Before and After Incident Coronary Events. J Am Coll Cardiol. 2019;73:3041–50. 10.1016/j.jacc.2019.04.019 31221251

