# Supplementary material for: Combined effects of cumulative triglyceride-glucose and blood pressure on stroke in middle-aged and older Chinese adults: a longitudinal analysis
Source: J Glob Health. 2026 May 22;16:04113. doi: 10.7189/jogh.16.04113 (PMC13196493; doi:10.7189/jogh.16.04113)
Supplement: Online Supplementary Document [file jogh-16-04113-s001.pdf]

Supplement to: Wang X, Zheng W, Jin X, Liu H, Hao J, Zhang L, Gu Y, Hou H, Wang W. Combined effects of cumulative triglyceride-glucose and blood pressure on stroke in middle-aged and older Chinese adults: a longitudinal analysis. J Glob Health. 2026;16:04113.

Abbreviations

AUC – area under the curve, BP – blood pressure, CCDC – Chinese Centre for Disease Control and Prevention, CHARLS – China Health and Retirement Longitudinal Study, CI – confidence interval, CumDBP – cumulative diastolic blood pressure, CumSBP – cumulative systolic blood pressure, CumTyG – cumulative triglyceride-glucose index, DBP – diastolic blood pressure, ENET – elastic net, FBG – fasting blood glucose, FDR – false discovery rate, HbA1c – glycosylated hemoglobin, HDL – high-density lipoprotein cholesterol, IQR – interquartile range, IR – insulin resistance, LDL – low-density lipoprotein cholesterol, MAPK – mitogen-activated protein kinase, OR – odds ratio, PI3K – phosphatidylinositol 3-kinase, ROC – receiver operating characteristic, SBP – systolic blood pressure, SD – standard deviation, Sp – specificity, Se – sensitivity, TC – total cholesterol, TG – triglyceride, TyG – triglyceride-glucose index, XGBoost – extreme gradient boosting

Figure S1 Logistic regression analysis for the association between co-exposure to CumTyG and CumBP on stroke incidence.

Figure S2 Feature selection result graphs after computerized process through machine learning methods involving Boruta (A, B), XGBoost (C) , ENET (D).

Figure S3 Identifying consensus factors through Venndiagram.

Figure S4 ROC curves of the machine learning algorithms.

Table S1 Baseline characteristics of study participants.

Table S2 Pearson correlation between TyG and SBP in 2011 and 2015.

Table S3 Pearson correlation between TyG and DBP in 2011 and 2015.

Table S4 Multivariable adjusted cross-lagged standard regression coefficients of TyG and SBP (n=5,138).

Table S5 Multivariable adjusted cross-lagged standard regression coefficients of TyG and DBP (n=5,138).

Table S6 Stratified analysis of stroke risk based on CumTyG and CumSBP.

Table S7 Stratified analysis of stroke risk based on CumTyG and CumDBP.

Table S8 Subgroup analysis between co-exposure to CumTyG and CumSBP on stroke incidence.

Table S9 Subgroup analysis between co-exposure to CumTyG and CumDBP on stroke incidence.

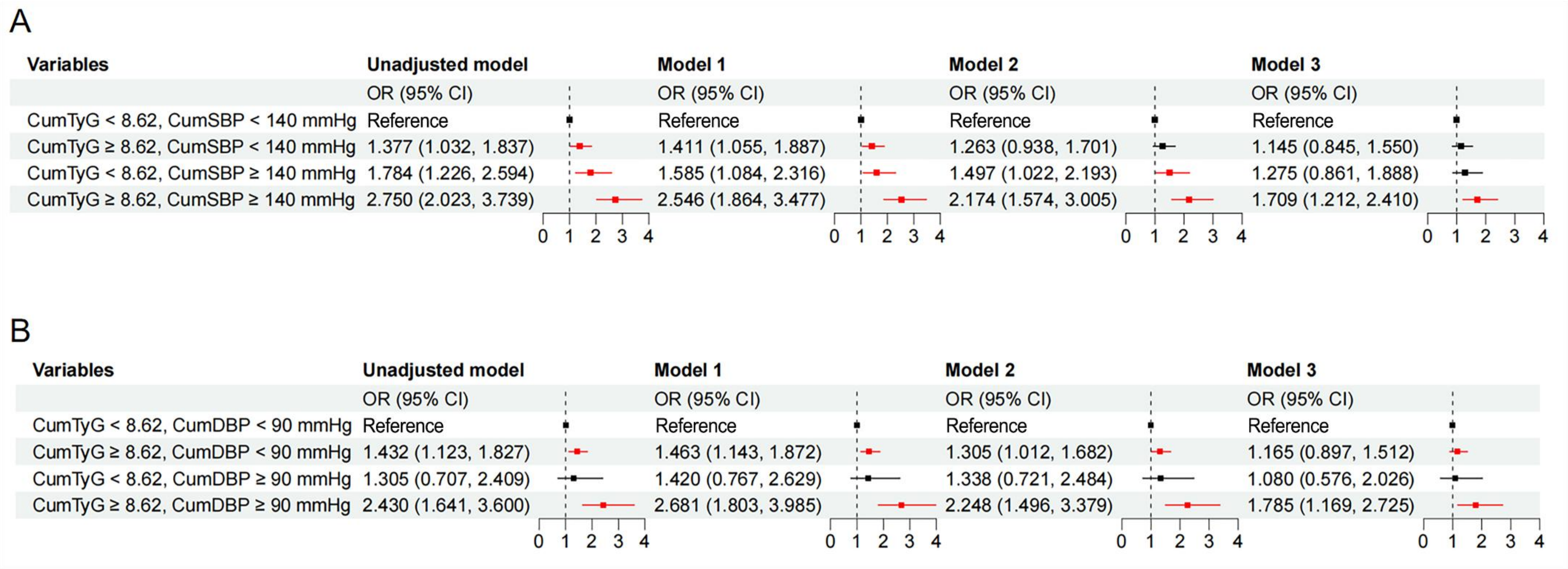

Figure S1 Logistic regression analysis for the association between co-exposure to CumTyG and CumBP on stroke incidence.

Panel A. Association between co-exposure to CumTyG and CumSBP and stroke incidence. **Panel B.** Association between co-exposure to CumTyG and CumDBP and stroke incidence. Model 1: adjusted for age and sex; Model 2: adjusted for age, sex, education level, smoking status, alcohol drinking and BMI; Model 3: adjusted for factors in model 2 and history of hypertension, dyslipidemia, diabetes, heart disease, antihypertensive, antidiabetic and lipid-lowering treatments.

BMI – body mass index, BP – blood pressure, CI – confidence interval, CumDBP – cumulative diastolic blood pressure, CumSBP – cumulative systolic blood pressure, CumTyG – cumulative triglyceride-glucose index, OR – odds ratio

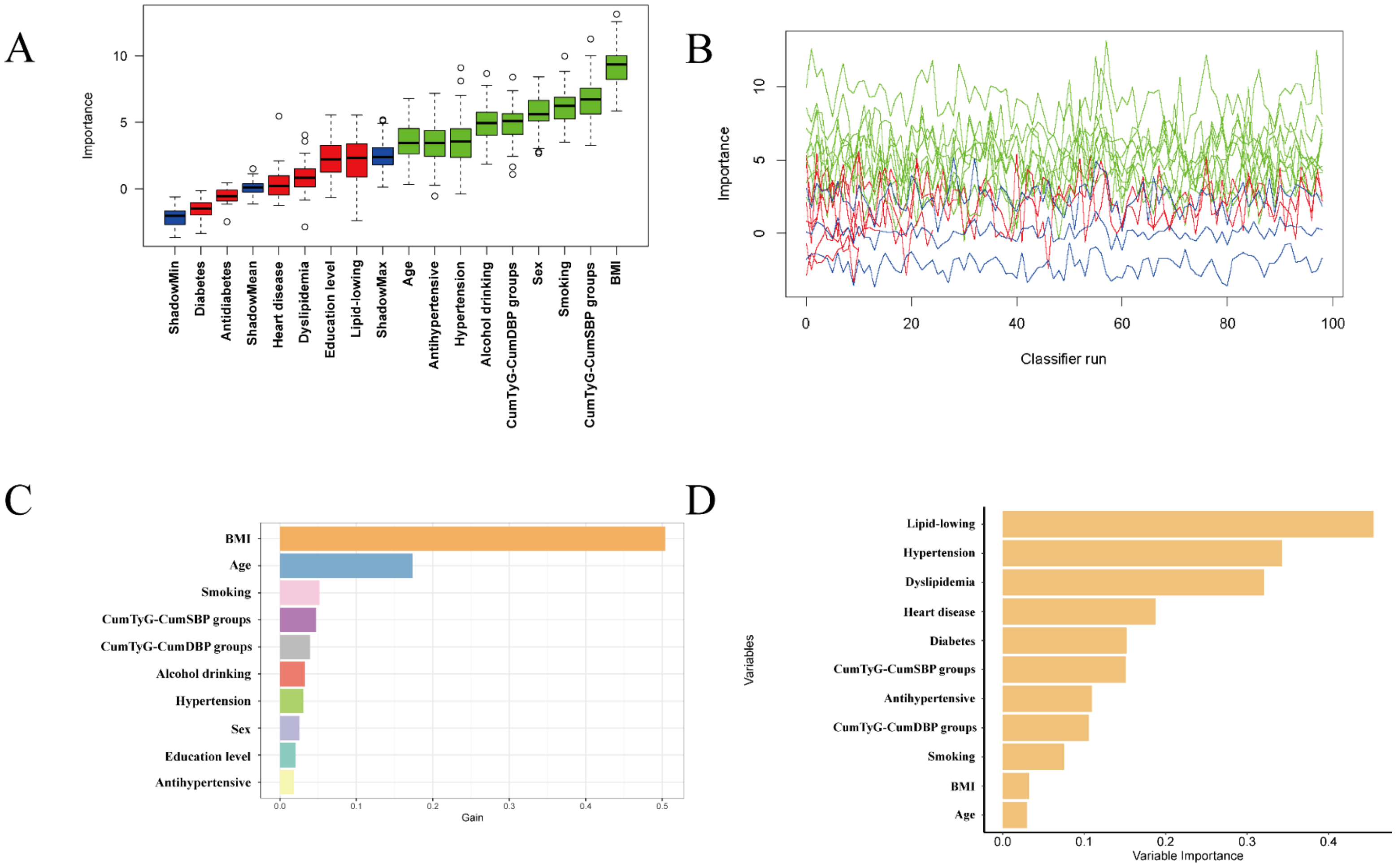

Figure S2 Feature selection result graphs after computerized process through machine learning methods involving Boruta (A, B), XGBoost (C) , ENET (D).

Panel A. Feature selection based on the Boruta algorithm. The horizontal axis is the name of each variable, and the vertical axis is the Z value of each variable. The box plot shows the Z value of each variable during model calculation. The green boxes represent important variables, and the red boxes represent unimportant variables. **Panel B.** The iteration process of Boruta algorithm. **Panel C.** The top 10 features sorted by importance based on XGBoost algorithm. Top-ranked variables indicate higher importance. **Panel D.** Key risk factor selection via ENET model.

BMI – body mass index, CumDBP – cumulative diastolic blood pressure, CumSBP – cumulative systolic blood pressure, CumTyG – cumulative triglyceride-glucose index, ENET – elastic net, XGBoost – extreme gradient boosting

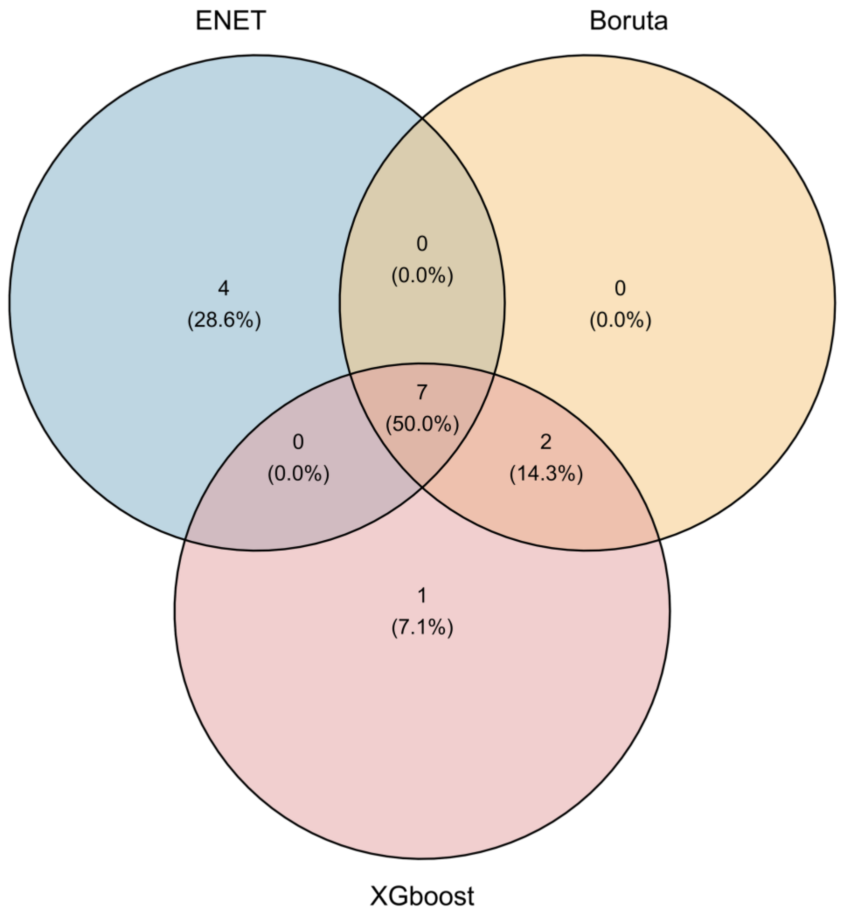

Figure S3 Identifying consensus factors through Venndiagram.

ENET – elastic net, XGBoost – extreme gradient boostin

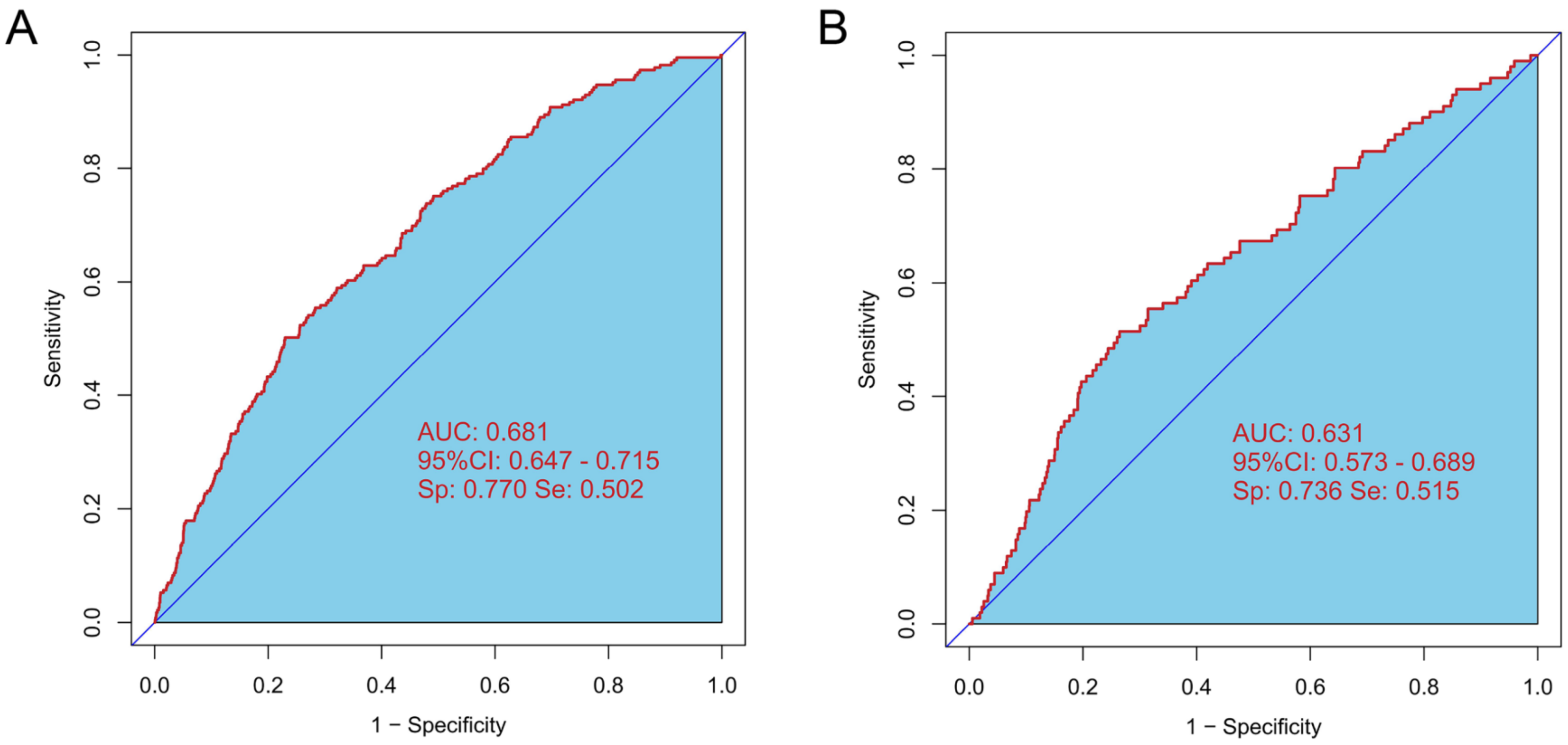

Figure S4 ROC curves of the machine learning algorithms.

Panel A. The training set ROC curve. **Panel B.** The validation set ROC curve.

AUC – area under the curve, ROC – receiver operating characteristic, Se – sensitivity, Sp – specificity

Table S1 Baseline characteristics of study participants.

Characters		Total (n=5138)	Non-stroke (n=4808)	Stroke (n=330)	*P*
Age, years		58.00 (52.00, 64.00)	58.00 (52.00, 64.00)	60.00 (55.00, 66.00)	<0.001
Sex (male, %)		2287 (44.51)	2142 (44.55)	145 (43.93)	0.829
Education level (n, %)	Primary and below	3618 (70.42)	3370 (70.09)	248 (75.15)	0.075
	Middle school	1461 (28.43)	1380 (28.70)	81 (24.55)	
	College and above	59 (1.15)	58 (1.21)	1 (0.30)	
Current married (n, %)		5093 (99.12)	4767 (99.15)	326 (98.79)	0.498
Smoking status (n, %)	Never	3191 (62.10)	2997 (62.33)	194 (58.79)	0.003
	Former	415 (8.08)	372 (7.74)	43 (13.03)	
	Current	1532 (29.82)	1439 (29.93)	93 (28.18)	
Alcohol drinking (n, %)	Never	3469 (67.52)	3239 (67.37)	230 (69.70)	0.586
	Former	411 (8.00)	384 (7.99)	27 (8.18)	
	Current	1258 (24.48)	1185 (24.65)	73 (22.12)	
BMI		23.24 (21.01, 25.89)	23.20 (20.97, 25.79)	23.92 (21.66, 26.85)	<0.001
TyG in 2011		8.60 (8.22, 9.05)	8.59 (8.21, 9.04)	8.73 (8.39, 9.12)	<0.001
SBP in 2011		129.00 (116.00, 144.00)	128.00 (116.00, 143.00)	136.00 (121.00, 152.00)	<0.001
DBP in 2011		75.00 (68.00, 84.00)	75.00 (68.00, 84.00)	78.00 (71.00, 87.25)	<0.001
CumTyG		8.62 (8.29, 9.04)	8.61 (8.28, 9.03)	8.74 (8.41, 9.16)	<0.001
CumSBP		127.50 (116.00, 140.50)	127.00 (115.50, 140.00)	134.75 (123.38, 148.63)	<0.001
CumDBP		75.00 (68.50, 82.00)	74.50 (68.00, 82.00)	77.50 (71.88, 84.63)	<0.001
Hypertension (n, %)		1229 (23.92)	1087 (22.61)	142 (43.03)	<0.001
Diabetes (n, %)		287 (5.59)	251 (5.22)	36 (10.91)	<0.001
Dyslipidemia (n, %)		490 (9.54)	424 (8.82)	66 (20.00)	<0.001

Antihypertensive treatments (n, %)	930 (18.10)	815 (16.95)	115 (34.85)	<0.001
Antidiabetic Treatments (n, %)	188 (3.66)	161 (3.35)	27 (8.18)	<0.001
Lipid-lowering treatments (n, %)	293 (5.70)	250 (5.20)	43 (13.03)	<0.001
Heart Disease (n, %)	567 (11.04)	506 (10.52)	61 (18.48)	<0.001
FBG	102.24 (94.45, 112.50)	102.24 (94.32, 112.32)	104.13 (95.72, 115.42)	0.012
TG	105.32 (74.34, 154.21)	104.43 (74.34, 153.10)	120.36 (84.96, 167.49)	<0.001
TC	190.59 (167.01, 215.34)	190.21 (167.01, 214.95)	195.04 (170.30, 219.98)	0.052
HDL	49.48 (40.59, 59.92)	49.48 (40.59, 59.92)	46.97 (39.05, 55.67)	<0.001
LDL	113.66 (93.17, 136.86)	113.66 (93.56, 136.47)	115.98 (92.40, 140.43)	0.276
HbA1c	5.10 (4.90, 5.40)	5.10 (4.90, 5.40)	5.15 (4.90, 5.50)	0.233

BMI – body mass index, CumDBP – cumulative diastolic blood pressure, CumSBP – cumulative systolic blood pressure, CumTyG – cumulative triglyceride-glucose index, DBP – diastolic blood pressure, FBG – fasting blood glucose, HbA1c – glycosylated hemoglobin A1c, HDL – high-density lipoprotein cholesterol, LDL – low-density lipoprotein cholesterol, SBP – systolic blood pressure, TC – total cholesterol, TG – triglyceride, TyG – triglyceride-glucose index

Table S2 Pearson correlation between TyG and SBP in 2011 and 2015.

**Variables**	**TyG 2011**	**SBP 2011**	**TyG 2015**	**SBP 2015**
TyG 2011	1.000	-	-	-
SBP 2011	0.075*	1.000	-	-
TyG 2015	0.479*	0.063*	1.000	-
SBP 2015	0.061*	0.487*	0.065*	-

Adjusted for age, sex, education level, smoking status, alcohol drinking, BMI, TC, antihypertensive, antidiabetic and lipid-lowering treatments; *$P < 0.001$; BMI – body mass index, SBP – systolic blood pressure, TC – total cholesterol, TyG – triglyceride-glucose index

Table S3 Pearson correlation between TyG and DBP in 2011 and 2015.

Variables	TyG 2011	DBP 2011	TyG 2015	DBP 2015
**TyG 2011**	1.000	-	-	-
**DBP 2011**	0.064*	1.000	-	-
**TyG 2015**	0.479*	0.068*	1.000	-
**DBP 2015**	0.057*	0.447*	0.080*	-

Adjusted for age, sex, education level, smoking status, alcohol drinking, BMI, TC, antihypertensive, antidiabetic and lipid-lowering treatments; *P < 0.001; BMI – body mass index, DBP – diastolic blood pressure, TC – total cholesterol, TyG – triglyceride-glucose index

Table S4 Multivariable adjusted cross-lagged standard regression coefficients of TyG and SBP (n=5,138).

**Variables**	**Crude mode**	***P***	**Model 1**	***P***	**Model 2**	***P***
R^2 of TyG	0.322	<0.001	0.317	<0.001	0.307	<0.001
R^2 of SBP	0.312	<0.001	0.305	<0.001	0.295	<0.001
TyG2011 to TyG2015	0.557 (0.538, 0.576)	<0.001	0.526 (0.505, 0.546)	<0.001	0.497 (0.475, 0.520)	<0.001
SBP2011 to SBP2015	0.549 (0.530, 0.569)	<0.001	0.526 (0.505, 0.547)	<0.001	0.508 (0.486, 0.530)	<0.001
TyG2011 to SBP2015	0.046 (0.023, 0.069)	<0.001	0.038 (0.014, 0.062)	0.002	0.026 (0.001, 0.051)	0.044
SBP2011 to TyG2015	0.052 (0.029, 0.075)	<0.001	0.039 (0.015, 0.063)	0.001	0.028 (0.003, 0.053)	0.027

Model 1: adjusted for age, sex, education level, smoking status, alcohol drinking and BMI;

Model 2: adjusted for covariates in Model 1 and TC, antihypertensive, antidiabetic and lipid-lowering treatments.

BMI – body mass index, SBP – systolic blood pressure, TC – total cholesterol, TyG – triglyceride-glucose index

Table S5 Multivariable adjusted cross-lagged standard regression coefficients of TyG and DBP (n=5,138).

**Variables**	**Crude mode**	***P***	**Model 1**	***P***	**Model 2**	***P***
R^2 of TyG	0.324	<0.001	0.317	<0.001	0.307	<0.001
R^2 of DBP	0.244	<0.001	0.247	<0.001	0.243	<0.001
TyG2011 to TyG2015	0.555 (0.536, 0.574)	<0.001	0.526 (0.506, 0.547)	<0.001	0.497 (0.475, 0.519)	<0.001
DBP2011 to DBP2015	0.487 (0.466, 0.508)	<0.001	0.470 (0.448, 0.492)	<0.001	0.456 (0.433, 0.479)	<0.001
TyG2011 to DBP2015	0.042 (0.018, 0.066)	0.001	0.033 (0.008, 0.057)	0.01	0.030 (0.004, 0.056)	0.023
DBP2011 to TyG2015	0.072 (0.050, 0.095)	<0.001	0.044 (0.021, 0.068)	<0.001	0.037 (0.013, 0.061)	0.002

Model 1: adjusted for age, sex, education level, smoking status, alcohol drinking and BMI;

Model 2: adjusted for covariates in Model 1 and TC, antihypertensive, antidiabetic and lipid-lowering treatments.

BMI – body mass index, DBP – diastolic blood pressure, TC – total cholesterol, TyG – triglyceride-glucose index

Table S6 Stratified analysis of stroke risk based on CumTyG and CumSBP.

Groups	Classifications	Model 1		Model 2	
		**OR (95%CI)**	***P***	**OR (95%CI)**	***P***
CumTyG < 8.62					
	CumSBP < 140 mmHg	Reference		Reference	
	CumSBP ≥ 140 mmHg	1.459 (0.987, 2.157)	0.058	1.274 (0.838, 1.937)	0.258
CumTyG ≥ 8.62					
	CumSBP < 140 mmHg	Reference		Reference	
	CumSBP ≥ 140 mmHg	1.744 (1.286, 2.366)	<0.001	1.507 (1.092, 2.078)	0.013
CumSBP < 140 mmHg					
	CumTyG < 8.62	Reference		Reference	
	CumTyG ≥ 8.62	1.192 (0.881, 1.614)	0.255	1.053 (0.770, 1.440)	0.745
CumSBP ≥ 140 mmHg					
	CumTyG < 8.62	Reference		Reference	
	CumTyG ≥ 8.62	1.614 (1.086, 2.399)	0.018	1.541 (1.028, 2.310)	0.036

Model 1: adjusted for age, sex, education level, smoking status, alcohol drinking and BMI; Model 2: adjusted for covariates in model 1 and history of hypertension, dyslipidemia, diabetes, heart disease, antihypertensive, antidiabetic and lipid-lowering treatments.

BMI – body mass index, CI – confidence interval, CumSBP – cumulative systolic blood pressure, CumTyG – cumulative triglyceride-glucose index, OR – odds ratio

Table S7 Stratified analysis of stroke risk based on CumTyG and CumDBP.

Groups	Classifications	Model 1		Model 2	
		OR (95%CI)	*P*	OR (95%CI)	*P*
CumTyG < 8.62					
	CumDBP < 90 mmHg	Reference		Reference	
	CumDBP ≥ 90 mmHg	1.293 (0.693, 2.411)	0.419	1.038 (0.543, 1.986)	0.910
CumTyG ≥ 8.62					
	CumDBP < 90 mmHg	Reference		Reference	
	CumDBP ≥ 90 mmHg	1.726 (1.167, 2.511)	0.006	1.520 (1.014, 2.277)	0.042
CumDBP < 90 mmHg					
	CumTyG < 8.62	Reference		Reference	
	CumTyG ≥ 8.62	1.313 (1.017, 1.695)	0.037	1.196 (0.920, 1.554)	0.181
CumDBP ≥ 90 mmHg					
	CumTyG < 8.62	Reference		Reference	
	CumTyG ≥ 8.62	1.664 (0.824, 3.359)	0.156	1.738 (0.813, 3.716)	0.154

Model 1: adjusted for age, sex, education level, smoking status, alcohol drinking and BMI; Model 2: adjusted for covariates in model 1 and history of hypertension, dyslipidemia, diabetes, heart disease, antihypertensive, antidiabetic and lipid-lowering treatments.

BMI – body mass index, CI – confidence interval, CumDBP – cumulative diastolic blood pressure, CumTyG – cumulative triglyceride-glucose index, OR – odds ratio

Table S8 Subgroup analysis between co-exposure to CumTyG and CumSBP on stroke incidence.

Stratifications		Group 1	Group 2	Group 3	Group 4	*P* for interaction
Sex	Female	Reference	1.109 (0.737, 1.668)	1.351 (0.786, 2.322)[a]	1.270 (0.784, 2.058)[ab]	0.140
	Male	Reference	1.159 (0.732, 1.835)	1.172 (0.657, 2.092)	2.441 (1.490, 3.998)[abc]	
Age, Years	≥60	Reference	0.981 (0.622, 1.548)	1.445 (0.883, 2.365)	1.984 (1.253, 3.141)[ab]	0.952
	<60	Reference	1.233 (0.815, 1.865)[a]	1.009 (0.508, 2.004)	1.393 (0.818, 2.370)[a]	
BMI, kg/m^2	≥24	Reference	0.857 (0.545, 1.347)	0.734 (0.380, 1.417)	0.981 (0.598, 1.607)[b]	0.227
	<24	Reference	1.307 (0.864, 1.977)	1.825 (1.111, 2.997)[a]	2.763 (1.705, 4.476)[ab]	
Hypertension	Yes	Reference	1.183 (0.638, 2.192)	1.274 (0.656, 2.474)	1.659 (0.915, 3.006)	0.851
	No	Reference	1.123 (0.785, 1.606)	1.252 (0.744, 2.107)	1.762 (1.116, 2.784)[ab]	
Diabetes	Yes	Reference	0.730 (0.192, 2.779)	0.828 (0.127, 5.417)	1.238 (0.304, 5.048)	0.986
	No	Reference	1.148 (0.838, 1.573)	1.269 (0.847, 1.901)[a]	1.706 (1.192, 2.440)[ab]	
Dyslipidemia	Yes	Reference	1.042 (0.461, 2.357)	1.382 (0.489, 3.903)	1.098 (0.449, 2.682)	0.718
	No	Reference	1.153 (0.829, 1.605)	1.257 (0.818, 1.933)	1.877 (1.293, 2.724)[ab]	
Heart Disease	Yes	Reference	1.162 (0.542, 2.491)	1.105 (0.404, 3.021)	1.023 (0.425, 2.463)	0.588
	No	Reference	1.115 (0.799, 1.556)	1.276 (0.830, 1.961)[a]	1.893 (1.304, 2.748)[ab]	

Group1: CumTyG < 8.62, CumSBP < 140 mmHg; Group2: CumTyG ≥ 8.62, CumSBP < 140 mmHg; Group3: CumTyG < 8.62, CumSBP ≥ 140 mmHg; Group4: CumTyG ≥ 8.62, CumSBP ≥ 140 mmHg; All models were adjusted for age, sex, education level, smoking status, alcohol drinking, BMI, history of hypertension, dyslipidemia, diabetes, heart disease, antihypertensive, antidiabetic and lipid-lowering treatments; FDR correction: a, statistically significant compared with Group1; b, statistically significant compared with Group2; c, statistically significant compared with Group3. BMI – body mass index, CumSBP – cumulative systolic blood pressure, CumTyG – cumulative triglyceride-glucose index, FDR – false discovery rate

Table S9 Subgroup analysis between co-exposure to CumTyG and CumDBP on stroke incidence.

		**Group 1**	**Group 2**	**Group 3**	**Group 4**	***P* for interaction**
Sex	Female	Reference	0.994 (0.700, 1.411)	1.051 (0.429, 2.575)	1.576 (0.883, 2.812)[ab]	0.521
	Male	Reference	1.430 (0.969, 2.110)[a]	1.085 (0.444, 2.654)	1.942 (1.032, 3.653)[a]	
Age, Years	≥60	Reference	1.104 (0.775, 1.572)	0.953 (0.365, 2.489)	1.788 (0.961, 3.325)[a]	0.681
	<60	Reference	1.221 (0.825, 1.806)[a]	1.213 (0.523, 2.818)	1.832 (1.015, 3.307)[a]	
BMI, kg/m^2	≥24	Reference	0.896 (0.603, 1.331)	0.804 (0.300, 2.155)	1.422 (0.834, 2.425)[ab]	0.303
	<24	Reference	1.398 (0.990, 1.975)[a]	1.359 (0.596, 3.099)	2.024 (0.953, 4.299)	
Hypertension	Yes	Reference	1.073 (0.678, 1.699)	0.915 (0.381, 2.200)	1.907 (1.073, 3.388)	0.722
	No	Reference	1.221 (0.886, 1.680)	1.216 (0.480, 3.082)	1.300 (0.608, 2.776)	
Diabetes	Yes	Reference	0.977 (0.310, 3.075)	2.210 (0.222, 22.043)	3.215 (0.751, 13.757)[ab]	0.167
	No	Reference	1.182 (0.904, 1.546)[a]	0.969 (0.492, 1.910)	1.576 (0.994, 2.500)[a]	
Dyslipidemia	Yes	Reference	0.988 (0.477, 2.045)	2.267 (0.690, 7.444)	2.112 (0.787, 5.671)[ab]	0.079
	No	Reference	1.210 (0.915, 1.601)	0.714 (0.305, 1.669)	1.633 (1.006, 2.651)[a]	
Heart Disease	Yes	Reference	0.974 (0.506, 1.877)	1.026 (0.205, 5.135)	1.848 (0.699, 4.885)[a]	0.929
	No	Reference	1.202 (0.904, 1.598)[a]	1.087 (0.546, 2.162)	1.789 (1.114, 2.874)[a]	

Group1: CumTyG < 8.62, CumDBP < 90 mmHg; Group2: CumTyG ≥ 8.62, CumDBP < 90 mmHg; Group3: CumTyG < 8.62, CumDBP ≥ 90 mmHg; Group4: CumTyG ≥ 8.62, CumDBP ≥ 90 mmHg; All models were adjusted for age, sex, education level, smoking status, alcohol drinking, BMI, history of hypertension, dyslipidemia, diabetes, heart disease, antihypertensive, antidiabetic and lipid-lowering; FDR correction: a, statistically significant compared with Group1; b, statistically significant compared with Group2; c, statistically significant compared with Group3. BMI – body mass index, CumDBP – cumulative diastolic blood pressure, CumTyG – cumulative triglyceride-glucose index, FDR – false discovery rate
